# Features and mechanisms of propofol-induced protein kinase C (PKC) translocation and activation in living cells

**DOI:** 10.3389/fphar.2023.1284586

**Published:** 2023-11-07

**Authors:** Soma Noguchi, Taketoshi Kajimoto, Takuya Kumamoto, Masashi Shingai, Soshi Narasaki, Tomoaki Urabe, Serika Imamura, Kana Harada, Izumi Hide, Sigeru Tanaka, Yuhki Yanase, Shun-Ichi Nakamura, Yasuo M. Tsutsumi, Norio Sakai

**Affiliations:** ^1^ Department of Molecular and Pharmacological Neuroscience, Graduate School of Biomedical and Health Sciences, Hiroshima University, Hiroshima, Japan; ^2^ Division of Biochemistry, Department of Biochemistry and Molecular Biology, Kobe University Graduate School of Medicine, Kobe, Japan; ^3^ Department of Synthetic Organic Chemistry, Graduate School of Biomedical and Health Sciences, Hiroshima University, Hiroshima, Japan; ^4^ Department of Anesthesiology and Critical Care, Graduate School of Biomedical and Health Sciences, Hiroshima University, Hiroshima, Japan; ^5^ Department of Dental Anesthesiology, Graduate School of Biomedical and Health Sciences, Hiroshima University, Hiroshima, Japan; ^6^ Department of Pharmacotherapy, Graduate School of Biomedical and Health Sciences, Hiroshima University, Hiroshima, Japan

**Keywords:** protein kinase C (PKC), propofol, nuclear translocation of proteins, propofol derivatives, intracellular organelle, anesthetic

## Abstract

**Background and purpose:** In this study, we aimed to elucidate the action mechanisms of propofol, particularly those underlying propofol-induced protein kinase C (PKC) translocation.

**Experimental approach:** Various PKCs fused with green fluorescent protein (PKC-GFP) or other GFP-fused proteins were expressed in HeLa cells, and their propofol-induced dynamics were observed using confocal laser scanning microscopy. Propofol-induced PKC activation in cells was estimated using the C kinase activity receptor (CKAR), an indicator of intracellular PKC activation. We also examined PKC translocation using isomers and derivatives of propofol to identify the crucial structural motifs involved in this process.

**Key results:** Propofol persistently translocated PKCα conventional PKCs and PKCδ from novel PKCs (nPKCs) to the plasma membrane (PM). Propofol translocated PKCδ and PKCη of nPKCs to the Golgi apparatus and endoplasmic reticulum, respectively. Propofol also induced the nuclear translocation of PKCζ of atypical PKCs or proteins other than PKCs, such that the protein concentration inside and outside the nucleus became uniform. CKAR analysis revealed that propofol activated PKC in the PM and Golgi apparatus. Moreover, tests using isomers and derivatives of propofol predicted that the structural motifs important for the induction of PKC and nuclear translocation are different.

**Conclusion and implications:** Propofol induced the subtype-specific intracellular translocation of PKCs and activated PKCs. Additionally, propofol induced the nuclear translocation of PKCs and other proteins, probably by altering the permeability of the nuclear envelope. Interestingly, propofol-induced PKC and nuclear translocation may occur via different mechanisms. Our findings provide insights into the action mechanisms of propofol.

## Introduction

Protein kinase C (PKC) belongs to the serine-threonine kinase family, which is comprised of 10 subtypes (Takai et al., 1979; Huang et al., 1986; Ohno et al., 1988; Nakanishi and Exton, 1992; Hanks and Hunter, 1995; Newton, 2018). PKCs contain regulatory and catalytic domains (Newton, 2018). Based on the structure of their N-terminal regulatory domains, the PKC family is classified into three subfamilies: conventional PKC (cPKC), novel PKC (nPKC), and atypical PKC (aPKC) (Huang et al., 1986; Ohno et al., 1988; Ono et al., 1988; Nakanishi and Exton, 1992; Newton, 2018). Their regulatory domain consists of two domains, C1 and C2, which bind to lipids and Ca^2+^, respectively. C1 domain is divided into two subdomains: C1A and C1B. cPKCs (α, βⅠ, βⅡ, and γ) have both C1 and C2 domains and are regulated by both lipids and calcium. In contrast, nPKCs (*δ*, *ε*, *θ*, and *η*) are affected by lipids but not calcium because they have only C1 domains. aPKCs (ζ and ι/*λ*) only have the C1A domains, which are not sensitive to phorbol esters, typical PKC activators (due to the lack of the C1 domain), and are not regulated by calcium (due to the lack of the C2 domain) (Ono et al., 1988; Pu et al., 2006; Newton, 2018).

PKCs alter their localization upon stimulation with various stimuli including G-protein-coupled receptors (GPCRs) and phosphorylate substrates at localized sites. This phenomenon is called PKC translocation (Sakai et al., 1997; Shirai et al., 1998b; Shirai and Saito, 2002). In cPKCs, GPCR stimulation produces diacylglycerol (DG) and Ca^2+^, which translocate cPKCs from the cytoplasm to the plasma membrane (PM) and activates them there. Then, cPKCs phosphorylate their substrates in the PM (Shirai et al., 1998b). In addition to GPCR stimulation, PKCs are translocated by phorbol esters, which bind to the C1B domain and continuously translocate PKCs from the cytoplasm to the PM (Sakai et al., 1997; Shirai et al., 1998b; Shirai and Saito, 2002).

Various lipid mediators, other than DG that are involved in GPCR-stimulation, also induce PKC translocation and activation. For example, saturated and unsaturated fatty acids, which are excised from membrane lipids by phospholipase A1 and phospholipase A2, respectively, induce subtype-specific PKC translocation and activation (Shinomura et al., 1991; Kasahara and Kikkawa, 1995; Shirai et al., 1998a; Shirai et al., 1998b; Kashiwagi et al., 2002). Ceramide, produced from sphingomyelin by sphingomyelinase, also induces subtype-specific PKC translocation and activates PKC (Kajimoto et al., 2001; Kashiwagi et al., 2002). Therefore, features of PKC translocation depend on the PKC subtype and the lipid mediators that activate it.

Propofol (2,6-diisopropylphenol) is an intravenous anesthetic that is widely used to induce and maintain general anesthesia and sedation (Kay and Rolly, 1977). Propofol is thought to exert its anesthetic effect by binding to *γ*-aminobutyric acid type A (GABA_A_) receptors, resulting in inhibitory effects on the central nervous system (Irifune et al., 2003). Propofol affects not only GABA_A_ receptors but also other receptors, such as TRPA1 and HCN1 channels (Chen et al., 2009; Fischer et al., 2010; Tibbs et al., 2013; Tang et al., 2014; Bu et al., 2018). These facts support the hypothesis that anaesthetic agents exert their anesthetic effects by acting on membrane proteins, i.e. the “membrane protein hypothesis”. On the other hand, it has been proposed that the interaction between anaesthetic agents and cell membranes exerts various effects, including anaesthetic effects. This “membrane lipid hypothesis” has long been debated, even before membrane protein hypothesis was proposed (Lee, 1976; Urban et al., 2006; Kopp Lugli et al., 2009; Gray et al., 2013). It has recently been shown that anaesthetic drugs alter the status of membrane lipids, particularly lipid rafts, resulting in potassium ion channel activation and cell hyperpolarization (Pavel et al., 2020). This is a novel finding supporting the membrane lipid hypothesis. PKC, which is also activated by the lipid mediators produced from membrane lipids, may be an important factor when anesthetics exert their actions based on the membrane lipid hypothesis.

In addition to propofol’s sedative and anesthetic effects, it can cause side effects such as vascular pain and hypotension. The prolonged use of high-dose propofol can also cause a potentially fatal syndrome, called propofol infusion syndrome (PRIS) (Kam and Cardone, 2007). It has not yet elucidated what is the mechanism underlying these side effects. It is possible that PKCs may be involved in the exertion of these side effects of propofol.

Propofol activates purified PKCs in a dose-dependent manner (Hemmings and Adamo, 1994). Moreover, propofol modulates the functions of cardiomyocytes, vascular endothelial cells, and neurons via PKCs (Kurokawa et al., 2002; Kanaya et al., 2003; Lu et al., 2009; Li et al., 2021). In particular, propofol-activated PKC phosphorylates endothelial NO synthase (eNOS), possibly producing NO and dilating vessels, which may be involved in propofol-induced hypotension and vascular pain (Wang et al., 2010; Oliveira-Paula et al., 2022).

We previously reported that propofol activates many types of PKCs *in vitro* without the requirement for other PKC activators (Miyahara et al., 2018). Furthermore, we found that propofol translocates PKCs in a subtype-specific manner (Miyahara et al., 2018). Based on these results, propofol, like the PKC activator phorbol ester or lipid mediators, is predicted to bind directly to PKCs, inducing translocation and activation of PKCs. However, the detailed properties and mechanisms underlying propofol-induced PKC translocation remain unclear. In addition, whether propofol activates intracellular PKCs in living cells remains unknown.

Therefore, in this study, we profoundly investigated the features of subtype-specific propofol-induced PKC translocation by including PKC subtypes not addressed in previous studies. In addition, we measured PKC activation at local intracellular sites using PKC activation indicators. We also elucidated the mechanism of nuclear translocation of some PKC subtypes. Finally, we also synthesized several propofol derivatives and investigated their effects on PKC translocation to determine the structural motifs of propofol required for propofol-induced PKC translocation.

## Materials and methods

### Materials

Materials were obtained from the following sources: propofol and histamine from FUJI FILM Wako Pure Chemical (Osaka, Japan), 12-O-tetradecanoylphorbol 13-acetate (TPA), 2,6-diisopropylphenol, and leptomycin B (LMB) from Sigma Aldrich (St. Louis, MO, United States), BAPTA-AM from Dojinido (Kumamoto, Japan), and glass-bottom culture dishes from MatTek Corporation (Ashland, OR, United States). All other reagents and instruments were of analytical grade. Specifically, 4-allylpropofol (4APr), 4-(3-azido-2-hydroxypropyl)propofol (AHPPr), and 4-(3-azido-2-oxopropyl)propofol (AOPPr) were synthesized from propofol via Claisen-Cope rearrangement of *O*-allylpropfol (Supplementary Material).

### Plasmids

Expression plasmids for PKC-GFPs (PKCα-GFP, PKCδ-GFP, PKCη-GFP, and PKCζ-GFP) used in this study were constructed as previously described (Sakai et al., 1997; Shirai et al., 1998a; Kajimoto et al., 2001; Kashiwagi et al., 2002; Shirai et al., 2011). Expression plasmids for C kinase activity reporter (CKAR) and target-CKAR were constructed as previously described (Violin et al., 2003; Gallegos et al., 2006)). The expression plasmid for laminB1-EGFP was a kind gift from Professor Tashiro (Department of Cellular Biology, Research Institute for Radiation Biology and Medicine, Hiroshima University). To generate a plasmid that can express doublet and triplet tandem GFP, we subcloned GFP cDNA fragments into the multicloning sites of pEGFP-CI (Takara Bio, Mountain View, CA, United States). A GFP cDNA with BglII and HindII sites in its 5′- and 3′-ends, respectively (BglII-GFP-HindIII), was obtained via polymerase chain reaction using pEGFP-CI as a template. Similarly, a cDNA fragment with EcoRI and BamHI sites in its 5′- and 3′-ends (EcoRI-GFP BamHI), respectively, was generated. To obtain an expression plasmid expressing doublet tandem GFP (2xGFP), BglII-GFP-HindIII was subcloned into the BglII and HindII sites of pEGFP-C1, which was designated as p2xGFP. To produce a plasmid expressing triplet tandem GFP (3xGFP), EcoRI-GFP BamHI was subcloned into the EcoRI and BamHI sites of p2xGFP, which was designated as p3xGFP.

### Cell culture and transfection

HeLa cells were purchased from the Riken Cell Bank (Tsukuba, Japan) and cultured in the Dulbecco’s modified Eagle’s medium supplemented with 10% fetal bovine serum, penicillin (100 units/mL), and streptomycin (100 μg/mL) in a 5% CO_2_ humidified atmospheric incubator at 37°C. For transfection, expression plasmids were electroporated using a electroporator NEPA21 (NEPA GENE, Chiba, Japan), according to the recommended protocol. Briefly, 10 μg of plasmid was transfected into 2 × 10^6^ cells and the transfected cells were seeded into appropriate culture dishes (glass bottom dish or 60-mm dish).

### Observation of propofol-induced translocation of proteins

We observed propofol-induced translocation of proteins, including PKC-GFP, 2 days after transfection. The culture medium of HeLa cells expressing various proteins was replaced with normal HEPES buffer composed of 135 mM NaCl, 5.4 mM KCl, 1 mM MgCl2, 1.8 mM CaCl2, 5 mM HEPES, and 10 mM glucose, pH 7.3 (Miyahara et al., 2018). Propofol was diluted with normal HEPES buffer to a final concentration of 100 μM. The propofol solution was sonicated before application. Propofol-induced protein translocation was observed over time using a fluorescence microscope (BZ900, Keyence, Osaka, Japan) or confocal laser-scanning microscope (LSM 780, Carl Zeiss Jena, Germany).

Changes in nuclear and cytoplasmic fluorescence were analyzed by observing changes in the line profiles of the fluorescence signals that transversed the nucleus, as shown in Supplementary Figure S4A. The intensity of the fluorescence signals on the line was measured using ImageJ software (Ver.1.46r). The average fluorescence intensity of cytosol before propofol administration was set at 100%. The change in average fluorescence intensity of each GFP-fused protein in the nucleus and cytoplasm after propofol administration is represented as a graph.

### Treatment with leptomycin B (LMB)

LMB (10 nM) was applied to HeLa cells expressing various proteins 1 day after transfection. After 15 h with LMB, propofol-induced protein translocation was observed.

### Fluorescence resonance energy transfer (FRET) imaging

Cells expressing kinase activity reporters were rinsed once with, and imaged in, Hank’s balanced salt solution containing 1 mM Ca2+. Images were acquired on an Olympus IX71 microscope (Olympus, Tokyo, Japan) using an electron-multiplying CCD camera, Cascade II 512 (Photometrics, Tucson, AZ), controlled by the MetaFluor software (universal Imaging). The optical filters and mirrors were obtained from Chroma Technologies. Using a 6% neutral density filter, CFP and FRET images were obtained every 15 s through a 430/24-nm excitation filter, ET-ECFP/EYFP/mCherry dichroic mirror (89006), and a 470/24-nm emission filter (CFP) or 535/30-nm emission filter (FRET). YFP emission was also monitored as a control for photobleaching through a 490/20-nm excitation filter, an ET-ECFP/EYFP/mCherry dichroic mirror, and a 535/30-nm emission filter. Excitation and emission filters were switched on filter wheels (MAC5000 PS-System 73005020, Ludl Electronic Products). The integration times were 100 ms for CFP and FRET and 50 ms for YFP. Images were reanalyzed using the MetaFlour Analyst (universal Imaging). Whole cells were selected such that there was no net movement of the targeted reporter in or out of the selected region, and Metafluor Analyst was used to calculate the average FRET ratio within the selected region. The trace for each imaged cell was normalized to t = 0–2 min averaging the baseline value. The normalized C/Y emission ratios were combined from at least three independent experiments and represented as the average of these corrected values ± SEM.

### Western blotting analysis

Western blotting was performed as described previously (Taguchi et al., 2021). Briefly, cell lysate samples were separated on 7.5% sodium dodecyl sulfate-polyacrylamide gels and transferred onto polyvinylidene fluoride membranes. Membrane was blocked with 5% skim milk in PBS-T (0.03% Triton-X) and incubated with anti-GFP antibody (GF200, Nakalai Tesque, Kyoto, Japan, 1:2000 dilution) for 16 h at 4°C. The membrane was then incubated with a horseradish peroxidase-conjugated anti-mouse IgG antibody (Jackson ImmunoResearch, West Grove, PA, United States of America; diluted 1:10,000) at room temperature for at least 1 h. Immunoreactive bands were visualized using a chemiluminescence detection kit (Chemi-Lumi One; Nacalai Tesque). Band density was measured using a chemiluminescence image analyzer (EZ-Capture MG; ATTO, Tokyo, Japan).

## Results

### Propofol-induced PKC translocation

We previously reported that propofol induces subtype-specific translocation of various types of PKC using SH-SY5Y cells (Miyahara et al., 2018). In the present study, to examine the detailed properties and mechanisms of propofol-induced translocation, we used HeLa cells, in which our knowledge of PKC translocation and intracellular activation is increasing. HeLa cells were transfected with various PKC-GFP cDNAs, and 2 days later, propofol-induced PKC translocation was observed using fluorescence microscopy and confocal laser-scanning microscopy.

### Propofol-induced cPKC (PKCα) translocation in HeLa cells

**FIGURE 1:**
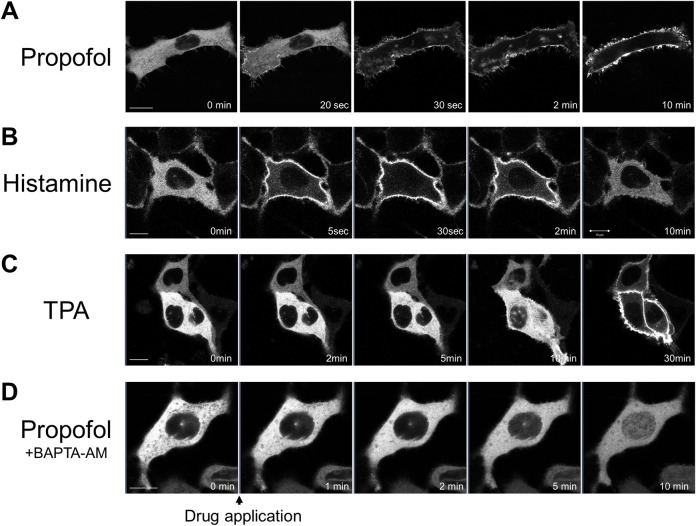
Sequential images of protein kinase C (PKC) fused with green fluorescent protein (PKC-GFP) translocation in HeLa cells elicited by various stimuli. Images were obtained using a confocal laser scanning microscope. **(A)** Application of 100 μM propofol elicited the translocation of PKCα-GFP to the plasma membrane (PM) within 1 min. PKCα-GFP was persistently localized in the PM during observation. Representative images of 28 observed cells are shown. **(B)** Application of 100 μM histamine elicited the translocation of PKCα-GFP to the PM within 30 s. PKCα-GFP almost completely returned to the cytoplasm within 5 min. Representative images of 12 observed cells are shown. **(C)** Application of 1 μM tetradecanoylphorbol 13-acetate (TPA) gradually and persistently translocated PKCα-GFP to the PM over 30-min of observation. Representative images of 12 observed cells are shown. **(D)** Propofol (100 μM) translocated PKCα-GFP to the nucleus in HeLa cells, in which intracellular calcium was eliminated by BAPTA-AM (10 μM). Representative images of 23 cells are shown. Bars indicate 10 μm.

We observed the translocation of PKCα (PKCα-GFP) in cPKC subtypes. PKCα is expressed in almost all tissues and regulates various cellular functions (Singh et al., 2017). PKCα translocated upon GPCR stimulation (Shirai and Saito, 2002). Histamine (100 μM) treatment rapidly translocated PKCα-GFP from the cytoplasm to the plasma membrane (PM), presumably via the histamine 1 receptor expressed in HeLa cells. PKCα-GFP returned to the cytoplasm within 5 min (Figure 1B). In contrast, treatment with propofol (100 μM) caused sustained translocation of PKCα-GFP from the cytoplasm to the PM for 8 min (Figure 1A). Tetradecanoylphorbol 13-acetate (TPA; 1 μM) caused sustained translocation of PKCα from the cytoplasm to the PM as previously reported (Shirai and Saito, 2002), but TPA more slowly translocated PKCα-GFP than propofol (Figure 1C). In addition, PKCα translocation is sensitive to Ca^2+^ via its Ca^2+^-binding C2 domain. To elucidate whether propofol-induced PKCα translocation depends on Ca^2+^, we buffered intracellular free Ca^2+^ by BAPTA-AM (10 μM). As a result, cytoplasmic PKCα-GFP translocated to the nucleus rather than the PM (Figure 1D). This finding suggests that propofol-induced translocation of PKCα to the PM depends on intracellular calcium levels.

### Propofol-induced translocation of nPKCs (PKCδ and PKCη) and aPKC (PKCζ) in HeLa cells

**FIGURE 2:**
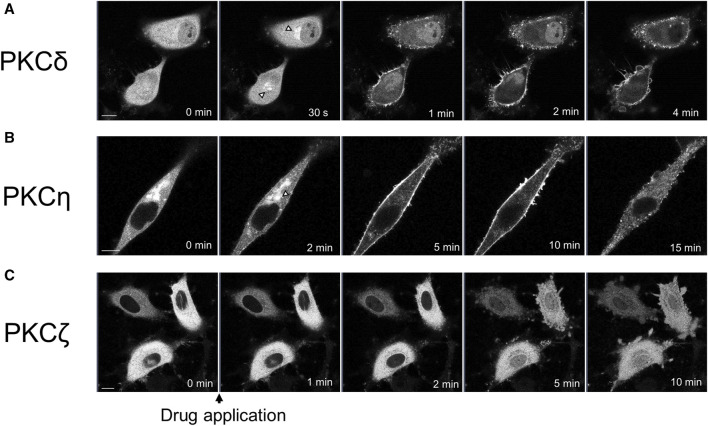
Sequential images of PKC translocation in HeLa cells induced by 100 μM propofol. Images were obtained using a confocal scanning microscope. **(A)** Application of propofol induced the translocation of PKCδ-GFP to the Golgi (arrowheads) within 30 s. PKCδ-GFP was persistently translocated to the PM during observation. Representative images of 32 cells are shown. **(B)** Application of propofol gradually translocated PKCη-GFP from the cytosol and Golgi (arrowhead) to the PM within 10 min. PKCη-GFP also accumulated in the intracellular dotted regions. Representative images of 15 cells are shown. **(C)** Propofol gradually translocated PKCζ-GFP to the nucleus during 10 min-observation. Representative images of 17 cells are shown. Bars indicate 10 μm.

We observed the translocation of PKCδ-GFP and PKCη-GFP, which are subtypes of nPKC. PKCδ is also distributed in almost all tissues (Steinberg, 2004). Treatment with propofol (100 μM) immediately translocated PKCδ-GFP from the cytoplasm to the Golgi and PM, 1 min after propofol treatment (Figure 2A).

PKCη is expressed mainly in the epidermis, gastrointestinal, and airway epithelia (Basu, 2019). PKCη-GFP predominantly localizes in the cytoplasm and Golgi. Upon treatment with propofol, PKCη-GFP was translocated to the PM or accumulated intracellularly in the punctate forms (Figure 2B). Simultaneous observation of PKCη-GFP with the ER tracker revealed that PKCη-GFP localization was consistent with that of endoplasmic reticulum (ER) both before and after propofol treatment (Supplementary Figure S2). As propofol (100 μM) changed the structure of ER as punctate forms, PKCη-GFP was also translocated to the accumulated puncta of ER (Supplementary Figure S2A). High-magnification images demonstrated that PKCη-GFP localized to the perinuclear and cytoplasmic ER before propofol treatment. Propofol facilitated the translocation of PKCη-GFP to the ER (Supplementary Figure S2B).

We observed translocation of PKCζ-GFP, a subtype of aPKC. PKCζ is expressed mainly in the brain, placenta, and adipocytes (Bandyopadhyay et al., 2002; Hapak et al., 2019). PKCζ-GFP began to translocate from the cytoplasm to the nucleus approximately 2 min after treatment with propofol, and by 5 or 10 min after treatment, the difference in fluorescence intensity between cytoplasmic and nuclear PKCζ-GFP almost disappeared (Figure 2C; Supplementary Figure S4B).

As shown in Figures 1, 2, various PKCs were translocated to the PM, Golgi, ER, and nucleus following propofol treatment in a subtype-specific manner. Translocation to the Golgi seems to be a common phenomenon in nPKC.

We investigated whether propofol at the concentration less than or more than 100 μM caused PKCs translocation to elucidate the threshold concentration for PKC translocation. Propofol (50 μM) did not induce the translocation of PKCs (Supplementary Figure S1A). Propofol at 200 and 100 μM elicited the translocation of all types of PKCs (Supplementary Figure S1B), indicating that the threshold concentration was between 50 and 100 μM, which corresponds to the threshold of propofol-induced intracellular calcium elevation at 75 μM (Urabe et al., 2020).

### Propofol-induced activation of PKC in subcellular localized regions

**FIGURE 3:**
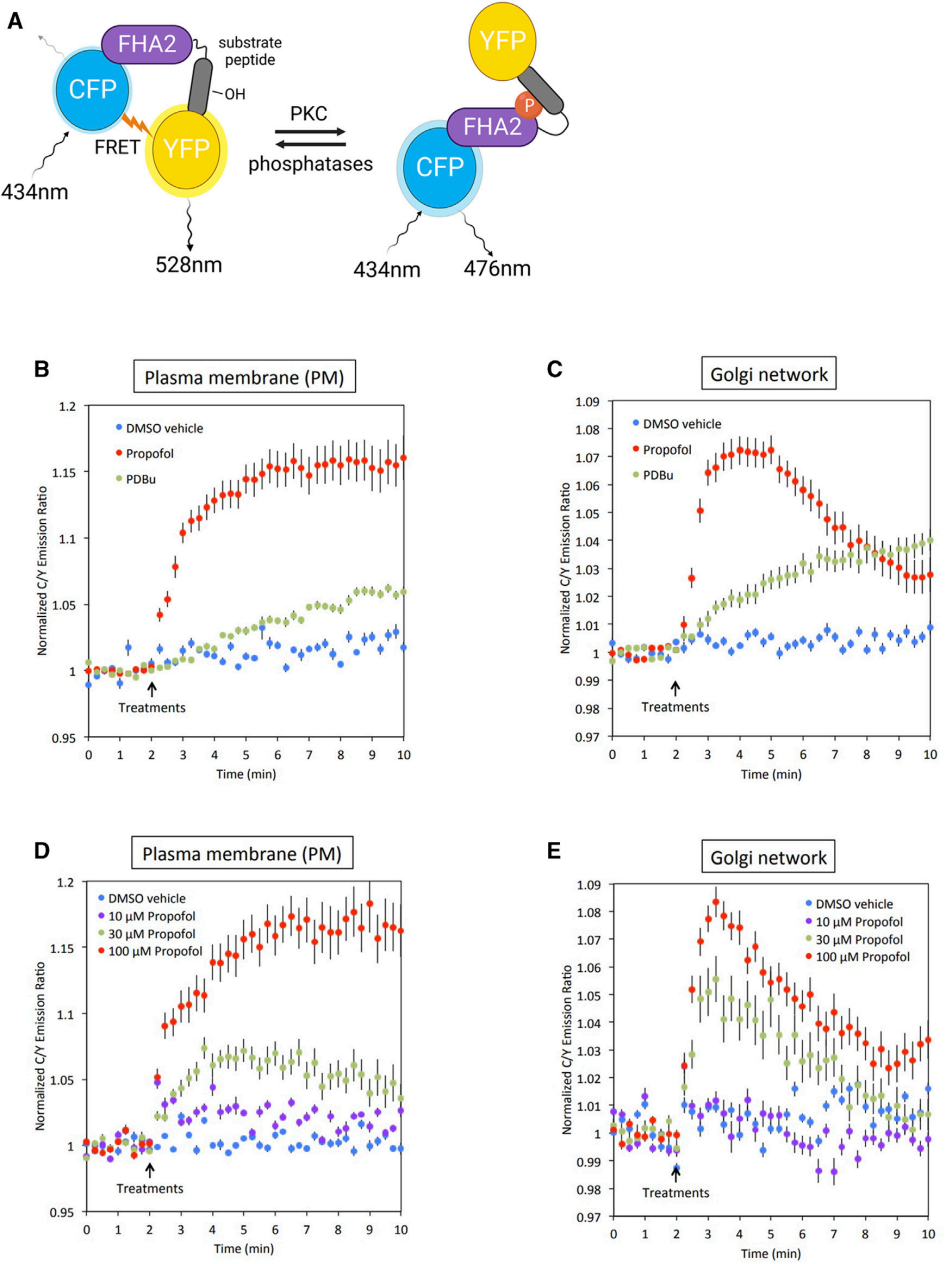
Conformation of PKC activation by propofol in living cells. **(A)** Principle of C kinase activity reporter (CKAR), a fluorescence resonance energy transfer (FRET)-based indicator of PKC activation. PM-CKAR and Golgi-CKAR were expressed in HeLa cells to monitor PKC activation in the PM and Golgi apparatus, respectively. **(B)** Application of 100 μM propofol robustly and persistently induced CKAR phosphorylation in the PM, which was estimated using cyan fluorescent protein (CFP)/yellow fluorescent protein (YFP) emission ratios. Propofol-induced PKC activation was more prominent than that induced by 200 nM PDBu. **(C)** Application of 100 μM propofol robustly induced CKAR phosphorylation in the Golgi apparatus. Phosphorylation of CKAR was transient compared to that observed in the PM. **(D)** Concentration-dependent PKC activation in the PM estimated using PM-CKAR. **(E)** Concentration-dependent PKC activation in the Golgi apparatus estimated using Golgi-CKAR. Bars indicate the mean ± standard error of the mean (SEM; n ≥ 23 cells).

In our previous study, we found that propofol activated various types of PKC *in vitro* (Miyahara et al., 2018). However, it remains unclear whether propofol activates PKCs and phosphorylates their substrates in the subcellular localized regions where PKC are translocated. To address this issue, we examined whether PKC was activated in the PM and Golgi, the main translocation sites of PKC, by targeting CKAR. CKAR is an intracellular indicator of PKC activation based on the FRET phenomenon. The FRET phenomenon occurs at a steady state and is lost when CKAR is phosphorylated by PKC (Figure 3A) (Violin et al., 2003). We assessed PKC activation in living cells by observing the FRET state of CKAR. Targeting CKARs (Gallegos et al., 2006), CKARs with signal peptides that induce organelle-specific expression, have been used (Gallegos et al., 2006). PM-CKAR and Golgi-CKAR were expressed in the PM and Golgi of HeLa cells, respectively.

### Activation of PKC by propofol in PM and Golgi

Propofol (100 μM) activated PKC in the PM and Golgi, the sites where PKC is translocated (Figures 3B, C). Propofol caused a more rapid and pronounced PKC activation than 200 nM PDBu, a phorbol ester PKC activator.

### Concentration-dependent activation of PKC by propofol

The cells were treated with propofol at concentrations ranging from 10 to 100 μM. As shown in Figures 3D, E, the propofol-induced activation of PKC was concentration-dependent. Notably, the results indicated that propofol at 30 μM, which corresponds to the blood concentration required for clinical use, sufficiently activated PKC in both the PM and Golgi.

**FIGURE 4:**
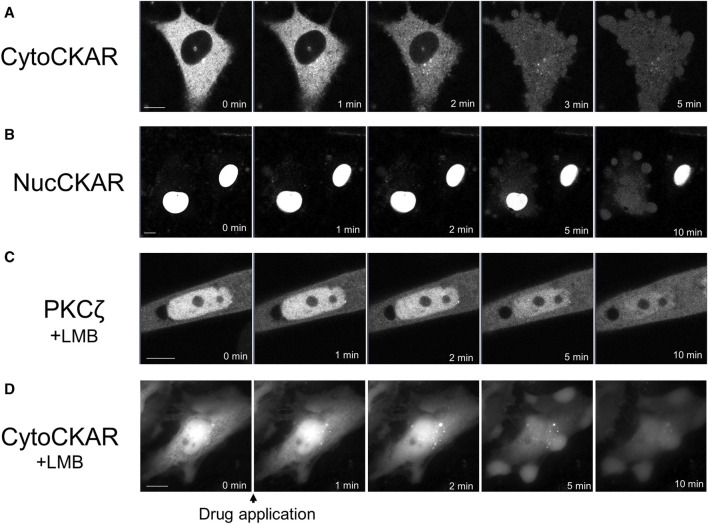
Propofol-induced translocation of proteins into and out of the nucleus. Various proteins shown in Figure 4 were expressed in HeLa cells and propofol (100 μM)-induced translocation of these proteins was observed. **(A)** Sequential images of translocation of Cyto-CKAR, a CKAR with nuclear export signal (NES), into the nucleus. **(B)** Sequential images of the translocation of Nuc-CKAR a CKAR with NLS, out of the nucleus. **(C)** PKCζ-GFP-expressing HeLa cells were treated with 10 nM leptomycin B (LMB), a CRM1 inhibitor, for 15 h before observation. Sequential translocation images revealed that nuclear-localized PKCζ-GFP was translocated to the cytosol until the fluorescence intensity inside and outside the nucleus became uniform. **(D)** Cyto-CKAR-expressing HeLa cells were treated with 10 nM LMB. Sequential translocation images showed that the nuclear-localized cyto-CKAR was translocated to the cytosol. Images in D were obtained using a fluorescence microscope. Bars indicate 10 μM. Images in Figure 4 are representative of 8-cells **(A)**, 8-cells **(B)**, 11-cells **(C)**, and 9-cells **(D)**. Bars indicate 10 μm.

PKC was translocated to the nucleus. Therefore, we attempted to determine the activation state of nuclear PKC using Nuc-CKAR. However, treatment with propofol leaked Nuc-CKAR out of the nucleus as will be shown later (Figure 4B). Thus, propofol-induced PKC activation in the nucleus could not be evaluated.

### Mechanism underlying the propofol-induced translocation of PKC to nucleus

Among propofol-induced PKC translocations, the translocations to the nucleus were observed in PKCζ and PKCα under conditions eliminating intracellular Ca^2+^. Propofol-induced translocation to the PM resulted in prominent PKC-GFP accumulation at that site, whereas PKC translocation to the nucleus resulted in the uniform fluorescence intensity of PKC-GFP inside and outside the nucleus (Supplementary Figure S4). These results suggest that propofol-induced PKC translocation to the nucleus occurs via a passive rather than an active mechanism.

### Propofol-induced nuclear translocation of proteins is not PKC-specific

To determine whether propofol-induced translocation to the nucleus was a PKC-specific phenomenon, we investigated the effect of propofol on the localization of proteins other than PKCs. We used Cyto-CKAR as a protein with nuclear export signals (NESs) and Nuc-CKAR as a protein with nuclear localization signals (NLSs) (Gallegos et al., 2006). First, cyto-CKAR was localized in the cytoplasm, but was translocated from the cytoplasm to the nucleus upon treatment with propofol (100 μM) (Figure 4A; Supplementary Figure S4C). In contrast, Nuc-CKAR was first localized in the nucleus, and propofol (100 μM) treatment translocated Nuc-CKAR from the nucleus to the cytoplasm (Figure 4B; Supplementary Figure S4D). These results indicate that propofol-induced translocation of proteins into and out of the nucleus is not a PKC-specific phenomenon.

### Propofol-induced nuclear translocation of proteins is not inhibited by leptomycin B (LMB)

We further investigated the mechanism underlying propofol-induced nuclear translocation of proteins. CRM1 (chromosome region maintenance 1) is involved in the nuclear export of proteins; CRM1 forms a complex with NES-bearing transport substrates and transports them from the nucleus to the nuclear exterior (Fung and Chook, 2014). LMB is an antifungal antibiotic (Hamamoto et al., 1983) that specifically covalently binds to CRM1 and inhibits the nuclear export of the protein (Kudo et al., 1999). To determine whether CRM1 is involved in propofol-induced nuclear translocation, we examined the effect of LMB, a CRM1 inhibitor, on protein nuclear translocation. If propofol-induced translocation was mediated by CRM1, it would not cause translocation in CRM1-treated cells. The cells were treated with 10 nM LMB for 15 h prior to observation. LMB treatment altered the localization of PKCζ and Cyto-CKAR, which were strongly localized in the nucleus but not in the cytoplasm (Figures 4C, D). Treatment with propofol resulted in translocation of both proteins from the nucleus to the cytoplasm (Figures 4C, D; Supplementary Figures S4E, F). These results indicate that CRM1 is not involved in propofol-induced nuclear translocation of proteins.

### Propofol-induced translocation of GFP

**FIGURE 5:**
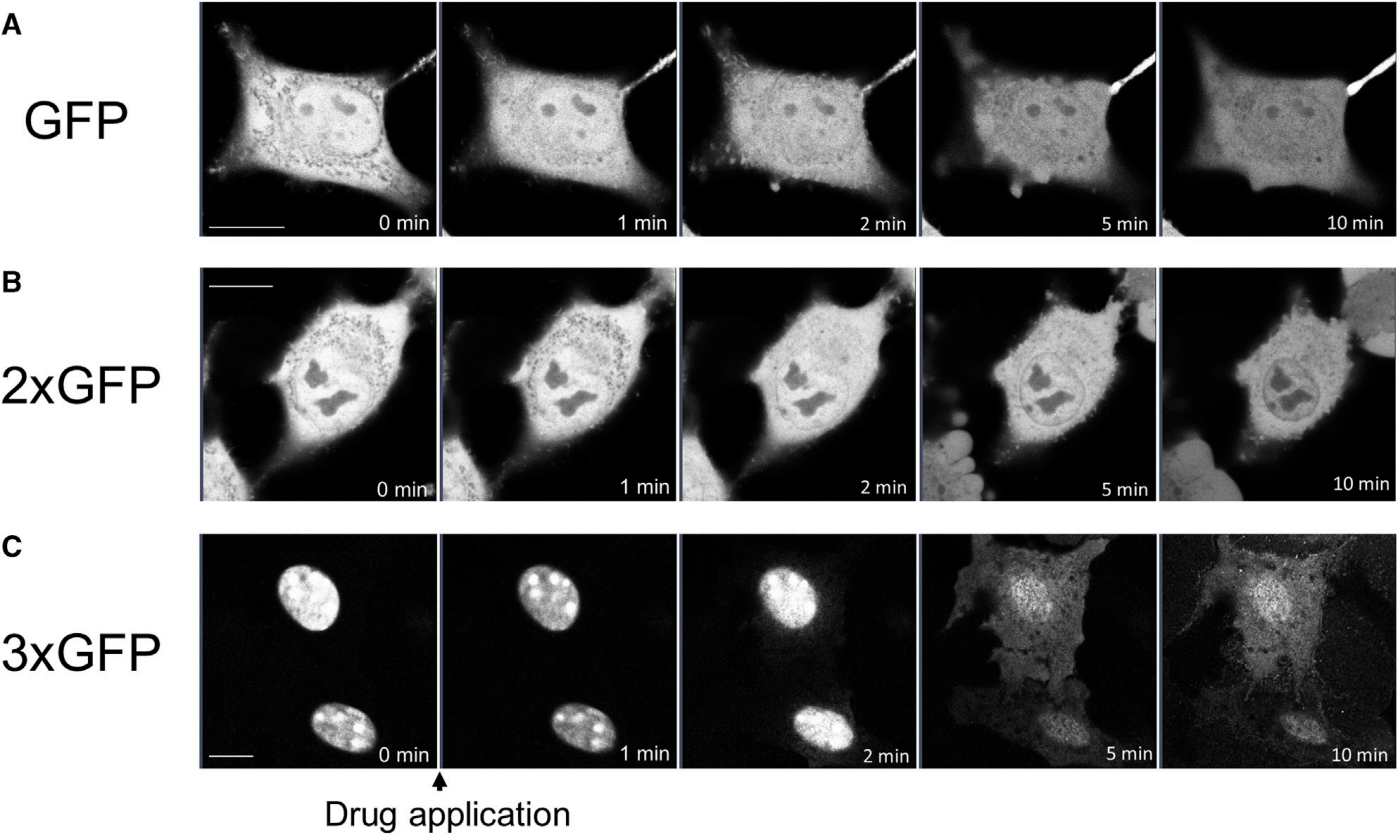
Propofol-induced translocation of singlet, doublet, and triplet GFP. Singlet GFP (GFP), doublet GFP (2xGFP) and triplet GFP (3xGFP) were expressed in HeLa cells, and propofol (100 μM)-induced translocation of these proteins was observed. **(A)** Sequential images of GFP translocation. GFP was uniformly expressed in the nucleus and cytoplasm before and after the application of 100 M propofol. **(B)** Sequential images of the translocation of 2xGFP; 2xGFP showed a slight tendency to be preferentially expressed in the nucleus. GFP fluorescence inside and outside the nucleus became uniform after the application of propofol. **(C)** Sequential images of translocation of 3xGFP; 3xGFP was clearly expressed in the nucleus. Propofol translocated 3xGFP to cytosol. Images in figure 5 are representative of 11-cells **(A)**, 5-cells **(B)**, and 12-cells **(C)** cells. Bars indicate 10 μm.

To determine whether propofol-induced translocation into and out of the nucleus was independent of NES or NLS, we examined the effects of propofol on GFP, which has neither NES nor NLS. Small molecules with a molecular weight of 40 kDa or less can move freely into and out of nucleus through the nuclear membrane pore complex (Lin and Hoelz, 2019). GFP molecules of approximately 27 kDa are unsuitable for observing propofol-induced localization changes because they can pass freely through this pore complex. Therefore, we expressed tandem GFPs, 2xGFP, and 3xGFP, which consisted of duplicate or triplicate GFPs linked together. Immunoblotting analysis revealed that these GFP and tandem GFPs had the appropriate molecular sizes (Supplementary Figure S3). GFP was uniformly expressed in the nucleus and cytoplasm of the HeLa cells (Figure 5A). 2xGFP showed a slight tendency to be preferentially expressed in the nucleus compared to the cytoplasm (Figure 5B; Supplementary Figure S4H). 3xGFP localized to the nucleus (Figure 5C), indicating that the addition of GFP allowed these tandem GFPs to localize to the nucleus. The expression of 2xGFP became uniform in and out of the nucleus within 10 min of treatment with propofol (100 μM) (Figure 5B; Supplementary Figure S4H). 3xGFP translocated from the nucleus to the cytoplasm, and its expression was almost uniform 5 min after propofol treatment (Figure 5C; Supplementary Figure S4I). 3xGFP, with a molecular size of 81 KDa is not expected to pass through the nuclear membrane pore complexes. Nevertheless, propofol translocated 3xGFP from the nucleus to the cytosol, suggesting that propofol altered the molecular permeability between the nucleoplasm and cytoplasm.

### Effects of propofol on the morphology of nucleus

**FIGURE 6:**
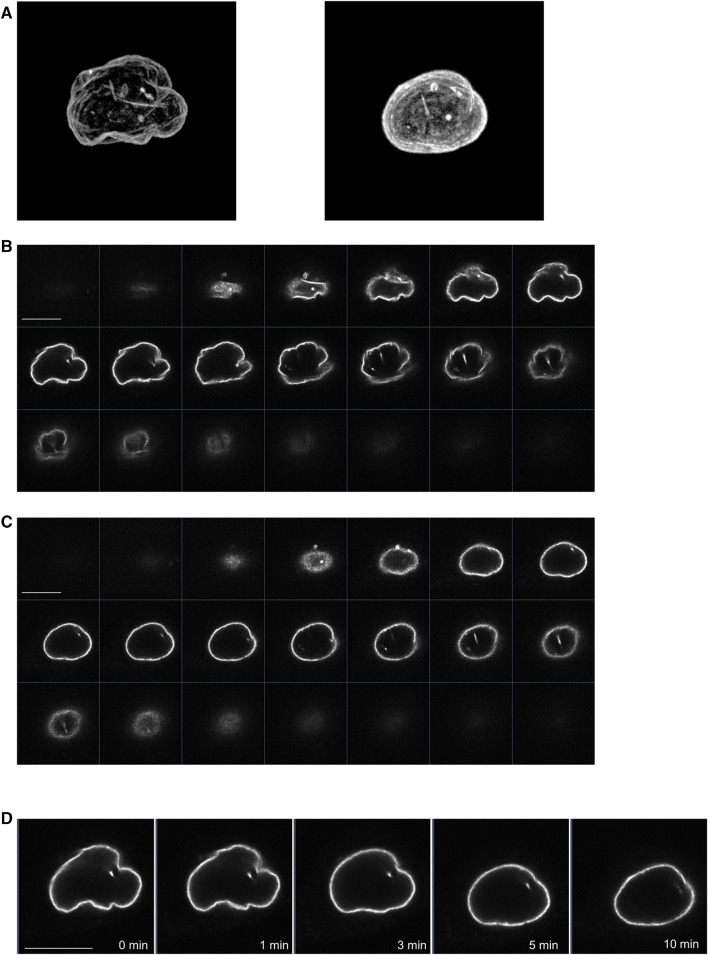
Propofol-induced morphological changes in the nucleus. LaminB1-EGFP was expressed in HeLa cells to observe the nuclear morphology. Propofol (100 μM)-induced morphological changes in the nucleus were observed using confocal laser scanning microscopy. **(A)**: left 3D-image of the nuclear envelope delineated by lamin-B1-EGFP before propofol treatment. Many wrinkles are observed. **(A)**: right 3D-image of nuclear envelope delineated by lamin-B1-EGFP after 10 min of propofol treatment. The wrinkles in the nucleus disappeared, and the nucleus became rounded. **(B)** Sequential images of lamin-B1-EGFP construct of the 3D-image of the nucleus in A. **(C)** Sequential images of lamin-B1-EGFP construct of the 3D-image of the nucleus in B. **(D)** Sequential time-lapse images of lamin-B1-EGFP after treatment with 100 μM propofol. The wrinkles in the nucleus disappeared, and the nucleus became rounded and shrunk slightly. Images in Figure 6 are representative of 7-cells. Bars indicate 10 μm.

We also examined whether propofol (100 μM) changed the morphology of the nucleus using LaminB1-EGFP, which can draw the nuclear morphology. Before treatment with propofol, the 3D-image of nucleus showed the wrinkles in the nuclear envelope (Figures 6A, C). After propofol treatment, the wrinkles in the nucleus disappeared, and the shape of the nucleus became rounded with slight shrinkage. However, prominent structural changes, such as rupture, did not occur because the distribution of LaminB1 was ubiquitous, and the nuclear envelope had no defects (Figures 6B, C, E).

### Structural motif of propofol involved in the propofol-induced PKC translocation

**FIGURE 7:**
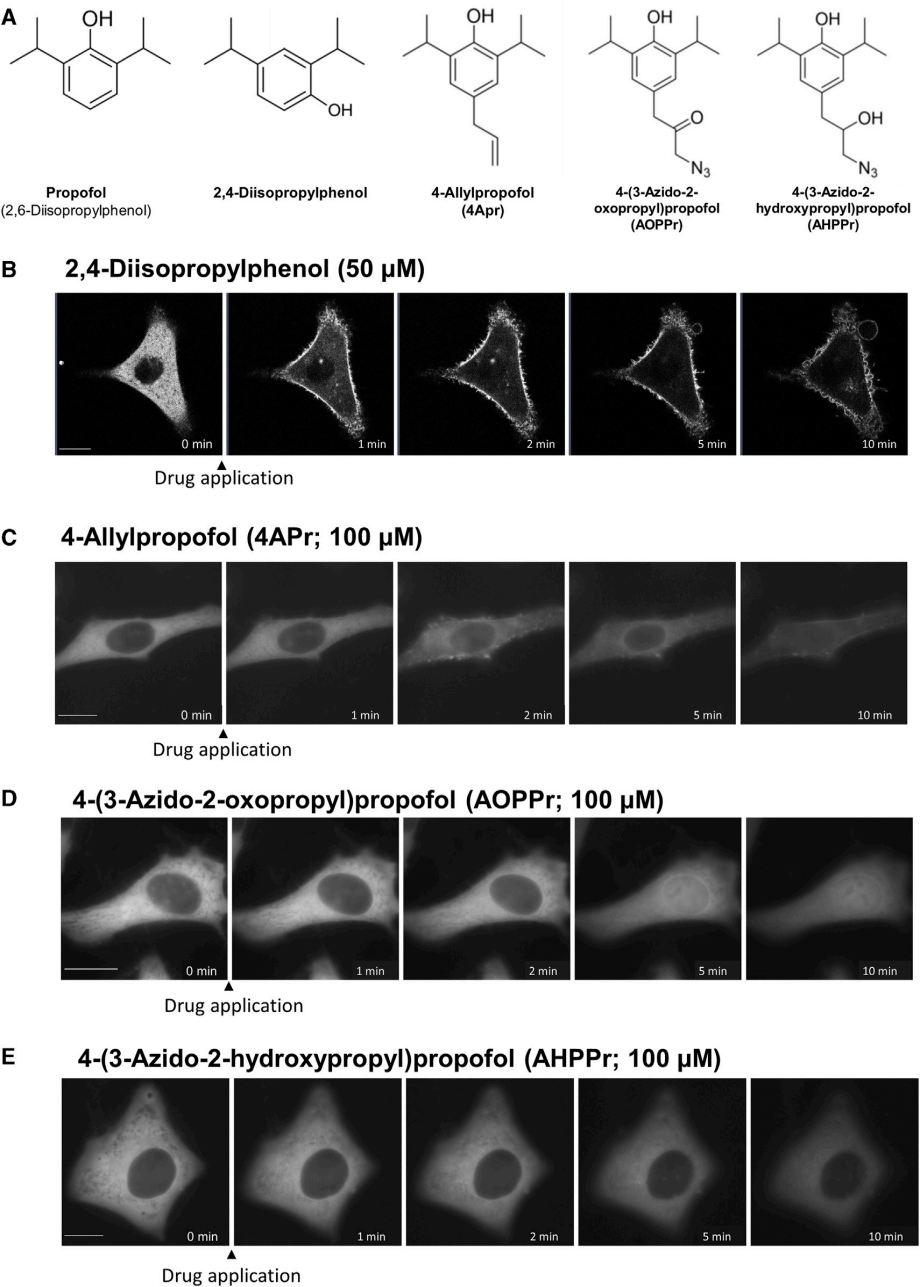
Effects of regioisomers and derivatives of propofol on PKCα translocation. **(A)** Structures of regioisomers and derivatives of propofol examined in this study. 2,4-Diisopropylphenol is a propofol regioisomer. 4-Allylpropofol (4APr), 4-(3-azido-2-oxopropyl)propofol (AOPPr), and 4-(3-azido -2-hydroxypropyl) propofol (AHPPr) are substituents at 4 position of propofol. **(B)** Sequential images of PKCα translocation induced by 50 μM 2,4-diisopropylphenol. **(C)** Sequential images of PKCα translocation induced by 100 μM 4-Allylpropofol (4APr). **(D)** Sequential images of PKCα translocation induced by 100 μM 4-(3-azido-2-oxopropyl)propofol (AOPPr). **(E)** Sequential images of PKCα translocation induced by 100 μM AHPPr. Images in Figure 7 are representative of 12-cells **(B)**, 4-cells **(C)**, 5-cells **(D)**, and 4-cells **(E)**. Bars indicate 10 μm.

To elucidate the important structural motif of propofol in the induction of PKC translocation and the transport of proteins into and out of the nucleus, we examined the effect of substituents at 4 position of propofol on PKC translocation (Figure 7).

2,4-Diisopropylphenol, a regioisomer of propofol, induced the translocation of PKCα and PKCδ similar to propofol (2,6-Diisopropylphenol) (Figure 7B; Supplementary Figure S5A), with a threshold concentration of 50 μM, which was less than that of propofol. We also designed and synthesized propofol derivatives bearing an azide group at the terminus of the propyl group at 4 position of propofol, which can be used for protein labeling. 4-Allylpropofol (4APr), a synthetic intermediate of azidepropofols, also exhibited similar properties for PKCα and PKCδ translocation as propofol (Figure 7C; Supplementary Figure S5B). In contrast, the effect of 4-(3-azido-2-oxopropyl) propofol (AOPPr) was remarkable; PKC was not translocated to the PM and Golgi, but to the nucleus (Figure 7D; Supplementary Figure S5C). It is supposed that AOPPr does not accumulate in the PM and Golgi and affects only the nuclear membrane. We also found that the addition of 4-(3-azido-2-hydroxypropyl) propofol (AHPPr) resulted in no translocation of PKC to the PM, Golgi, or nucleus, suggesting that PKC translocation is sensitive to substituents at 4 position in propofol derivatives. In addition, these findings suggest that the structural motif of propofol, which is involved in the induction of PKC translocation, differs from that involved in nuclear protein translocation.

## Discussion

### Character of subtype-specific PKC translocation

In this study, we examined the detailed mechanisms and properties of propofol-induced PKC translocation. PKCα translocated persistently from the cytoplasm to PM upon propofol treatment (Figure 1A). This PKCα translocation required an increase in intracellular calcium (Figure 1D). Since PKCγ also persistently translocates from the cytoplasm to the PM (Miyahara et al., 2018), this translocation property seen in PKCα is considered typical of propofol-induced translocation of cPKCs. In addition, GPCR-stimulated cPKC translocation was transient (Figure 1B), whereas propofol retained PKCα at the PM. Because propofol-induced increases in intracellular Ca^2+^ are transient (Urabe et al., 2020), propofol itself is required for PKC to persistently localize to the PM. Propofol localized in the PM may bind directly to PKC, thereby retaining PKC in the PM. In addition to cPKC, nPKC translocates from the cytoplasm to the PM. However, nPKC are translocated to specific intracellular organelles. PKCδ translocated to the Golgi (Figure 2A) and PKCη to the ER (Figure 2B). It has been shown that nPKC is translocated to Golgi by lipid mediators such as ceramide and arachidonic acid (Shirai et al., 1998a; Shirai et al., 1998b; Kashiwagi et al., 2002). Propofol, like a lipid mediator, can localize to the lipid membranes of intracellular organelles and trap cytoplasmic PKC, which can translocate PKC to specific intracellular organelles.

Propofol is expected to readily penetrate cells and reach various intracellular sites because of its low molecular weight and high lipophilicity. However, it remains difficult to visualize propofol directly and observe its detailed dynamics in cells at high resolution. However, a recent report on the visualization of propofol using stimulated Raman scattering microscopy showed that propofol is highly concentrated and localized at PM by using stimulated Raman scattering (SRS) microscopy (Oda et al., 2022). In addition, propofol causes morphological changes in the ER and mitochondria (Urabe et al., 2020) and possibly localizes to these intracellular organelles after its intracellular penetration.

PKCζ was translocated to the nucleus following propofol treatment (Figure 2C). Previous studies demonstrated that arachidonic acid translocates PKCζ of aPKC into the nucleus (Shirai et al., 1998b). We observed the propofol-induced translocation of nPKCs to the nucleus, PM, and intracellular organelles. Furthermore, nuclear translocation was also observed in PKCα under the condition of intracellular calcium removal (Figure 1D). Thus, the propofol-induced PKC translocation into the nucleus is not specific to PKCζ. In a later section, we discuss the mechanism underlying propofol-induced nuclear translocation of PKCs. In the present study, the threshold of propofol concentration at which PKC could translocate was between 50 and 100 µM. This concentration was almost identical to the concentration at which propofol causes intracellular calcium elevation and morphological changes in the ER (Urabe et al., 2020) but was apparently above the concentration at which propofol is used clinically. However, it is possible that the concentration reached the threshold for PKC translocation at the site of propofol injection.

### Propofol-induced activation of PKC at its translocated sites

However, it remains unclear whether propofol activates PKC by inducing its translocation in living cells. In this study, by targeting CKAR, we estimated PKC activation in the PM and Golgi, where PKC is translocated by propofol. The results showed that propofol caused rapid and significant PKC activation in a concentration-dependent manner in both the PM and Golgi (Figures 3B–E). The extent of activation by propofol was comparable to or more significant than that by phorbol ester (200 nM PDBu), a common PKC activator. In addition, even propofol at 30 μM, the blood concentration at clinical use (Kirkpatrick et al., 1988; Han et al., 2016), was sufficient to activate PKC, suggesting that propofol could activate PKC in case of clinical use. However, there is a gap in the thresholds for propofol concentration in PKC activation and translocation. Although the reason for this discrepancy is currently unknown, it is possible that localized and subtle translocations occur in the vicinity of the PM and Golgi, which cannot be captured by microscopic observation, in 30 μM propofol-treated cells. Alternatively, this discrepancy may be due to differences in the sensitivity of the detection methods used. That is, FRET may be a more sensitive assay than the measurement of fluorescence intensity.

Alternatively, PRIS is thought to be caused by prolonged exposure to propofol at higher concentrations than those in clinical use (Krajčová et al., 2015). We have shown that PKC activation and translocation are elicited by propofol of the order of 30–100 µM. This suggests that PKC may be involved in the manifestation of PRIS symptoms.

### Mechanism of propofol-induced PKC translocation into or out of the nucleus

Propofol translocated PKCζ to the nucleus (Figure 2C). Propofol also translocated PKCα to the nucleus in the absence of intracellular Ca^2+^ (Figure 1D). The difference in PKC-GFP fluorescence intensity inside and outside the nucleus became uniform after PKC was translocated into the nucleus (Supplementary Figure S4), unlike PKC translocation to the PM or intracellular organelles, where it clearly accumulates. These results suggest that propofol-induced PKC translocation to the nucleus occurs via a passive rather than an active mechanism. In addition, propofol altered the localization of several intranuclear and extranuclear proteins including Nuc-CKAR, cyto-CKAR, and PKC (Figure 4). These findings also indicate that propofol-induced nuclear translocation of the protein is not a specific phenomenon for PKCs. This uniform distribution of proteins inside and outside the nucleus was not followed by morphological changes in the nuclear envelope (Figure 6) and was independent of the NES and NLS (Figure 4). These results suggest that propofol alters the permeability of proteins that passively pass through the nuclear envelope, resulting in a uniform protein concentration in the nucleoplasm and cytoplasm. This idea is supported by the finding that propofol caused uniform intranuclear and extranuclear distribution of GFP, 2xGFP, and 3xGFP (Figure 5; Supplementary Figures S4G–I), which probably have the same properties with respect to localization but differ only in molecular weight.

These results suggest that propofol may alter the molecular permeability between the nucleoplasm and cytoplasm. This mechanism may explain why propofol induced a uniform distribution of PKC-GFP inside and outside the nucleus (Figures 1D, 2C). As these propofol-induced translocations preferably occur when exposed to high concentrations of propofol, this phenomenon may be involved in propofol-induced adverse events such as PRIS that occur with long-term administration of high concentrations of propofol.

### Structural motif of propofol involved in propofol-induced protein translocation

We further examined the effects of isomers and derivatives of propofol on protein translocation. We synthesized propofol derivatives with substituents at 4 position. Among them, 2,4-diisopropylphenol and 4APr induced the translocation of PKCα and PKCδ similar to propofol. In contrast, AOPPr translocated PKCα and PKCδ to the nucleus, but not either the PM or Golgi, suggesting that the structural motif of propofol, which is involved in the induction of PKC translocation to the PM and Golgi, is different from that involved in protein translocation into and out of the nucleus. At present, however, the possibility cannot be ruled out that these effects may be emerging due to differences in the proteins to which the isomers and derivatives of propofol bind.

### Limitations

In this study, propofol concentration used in most experiments was higher than the clinically used range, approximately below 30 µM. The concentration of propofol used in many experiments for PKC translocation was set at 100 µM because 100 µM propofol can reliably cause PKC translocation. Moreover, the duration of propofol treatment was only 15 min, which is a short period. Considering the possibility that lipophilic propofol accumulates in the lipid membranes of cells, further studies should investigate the translocation and activation of PKCs after the exposure of cells to propofol at lower concentrations for longer periods.

## Conclusion

In conclusion, propofol induces subtype-specific PKC translocation and activates it at the translocation site. Moreover, propofol-induced nuclear translocation of proteins is not PKC-specific. Notably, the translocation of protein into or out of the nucleus is independent of the mechanisms involved in NLS and NES and mediated by a passive mechanism. Our findings suggest that propofol-induced changes in the localization of PKC and other proteins may be involved in the induction of anesthesia or adverse effects of propofol.

## Data Availability

The original contributions presented in the study are included in the article/Supplementary Material, further inquiries can be directed to the corresponding author.

## Supplementary material

SUPPLEMENTARY FIGURE S1
**(A)** Sequential images of the translocation of green fluorescent protein (GFP)-fused protein kinase C (PKC)-α, PKCδ, PKCη, and PKCζ induced by 50 μM propofol. **(B)** Sequential images of the translocation of GFP-fused PKCα, PKCδ, PKCη, and PKCζ induced by 200 μM propofol. Images shown in Supplementary Figure S1 were obtained using a fluorescence microscope. Bars indicate 10 μm.

SUPPLEMENTARY FIGURE S2 Simultaneous observation of PKCη-GFP translocation and endoplasmic reticulum (ER) tracker dynamics induced by 100 μM propofol. Images were obtained using a confocal laser-scanning microscope. **(A)** Sequential images of propofol (100 μM)-induced PKCη-GFP translocation and ER-tracker dynamics. PKCη-GFP co-localized with the ER tracker before and after propofol application. Propofol altered the localization in ER as dots. PKCη-GFP was also translocated to the accumulated dots in the ER. **(B)** High-magnification images of PKCη-GFP and ER tracker colocalization. PKCη-GFP is abundantly expressed in the perinuclear ER. PKCη-GFP was also localized in the cytoplasmic reticular ER before the application of propofol (arrowheads, 0 s). Propofol translocated PKCη-GFP to the ER (arrowheads, 20 s). PKCη-GFP was translocated to the ER and accumulated as dots 3 min after application. Bars indicate 10 and 3 μm for A and B, respectively.

SUPPLEMENTARY FIGURE S3 Western blotting analysis of singlet GFP and tandem GFPs (2xGFP and 3xGFP). GFP and tandem GFPs were expressed in HeLa cells. Two days after transfection, cell lysates were prepared. Immunoblotting with anti-GFP antibody revealed the molecular sizes of singlet GFP, 2xGFP, and 3xGFP. Asterisks indicate singlet and tandem GFP bands.

SUPPLEMENTARY FIGURE S4 Time course of propofol-induced changes in the fluorescence intensity inside and outside the nucleus. **(A)** Fluorescence intensity of the line crossing the nucleus was measured using the ImageJ software (Ver.1.46r). Average of the cytoplasmic fluorescence intensity before propofol administration was set to 100%. **(B–F)** Time course of changes in the average nuclear and cytoplasmic fluorescence intensities of the respective GFP-fused proteins. Abscissa represents the time after propofol administration (min), and the ordinate represents the average fluorescence intensity in the cytoplasm and nucleus (%). Solid lines indicate the changes in fluorescence intensity in the nucleus, and dashed lines indicate the changes in fluorescence intensity in the cytoplasm.

SUPPLEMENTARY FIGURE S5 Sequential images of PKCδ-GFP translocation induced by 50 μM 2,4-diisopropylphenol **(A)**, 100 μM 4-allylpropofol (4APr) **(B)**, 100 μM 4-(3-azido-2-oxopropyl)propofol (AOPPr) **(C)**, and 100 μM 4-(3-azido-2-oxopropyl)propofol (AOPPr) **(D)**. Translocation of PKCδ-GFP induced by 2,4-diisopropylphenol and 4APr was similar to that induced by propofol. PKCδ-GFP first accumulated in the Golgi and then translocated to the PM. AOPPr did not translocate PKCδ-GFP to the Golgi and PM but translocated it to the nucleus, which is similar to the propofol-induced translocation of PKCζ-GFP and cyto-CKAR. AOPPr did not induce PKCδ-GFP translocation. Images were captured using a fluorescence microscope. Images in Supplementary Figure S5 indicate 2-cells **(A)**, 2-cells **(B)**, 5-cells **(C)**, and 2-cells **(D)**. Bars indicate 10 μm.
