# Supplementary figures and images for: Features and mechanisms of propofol-induced protein kinase C (PKC) translocation and activation in living cells
Source: Front Pharmacol. 2023 Nov 7;14:1284586. doi: 10.3389/fphar.2023.1284586 (PMC10662334; doi:10.3389/fphar.2023.1284586)

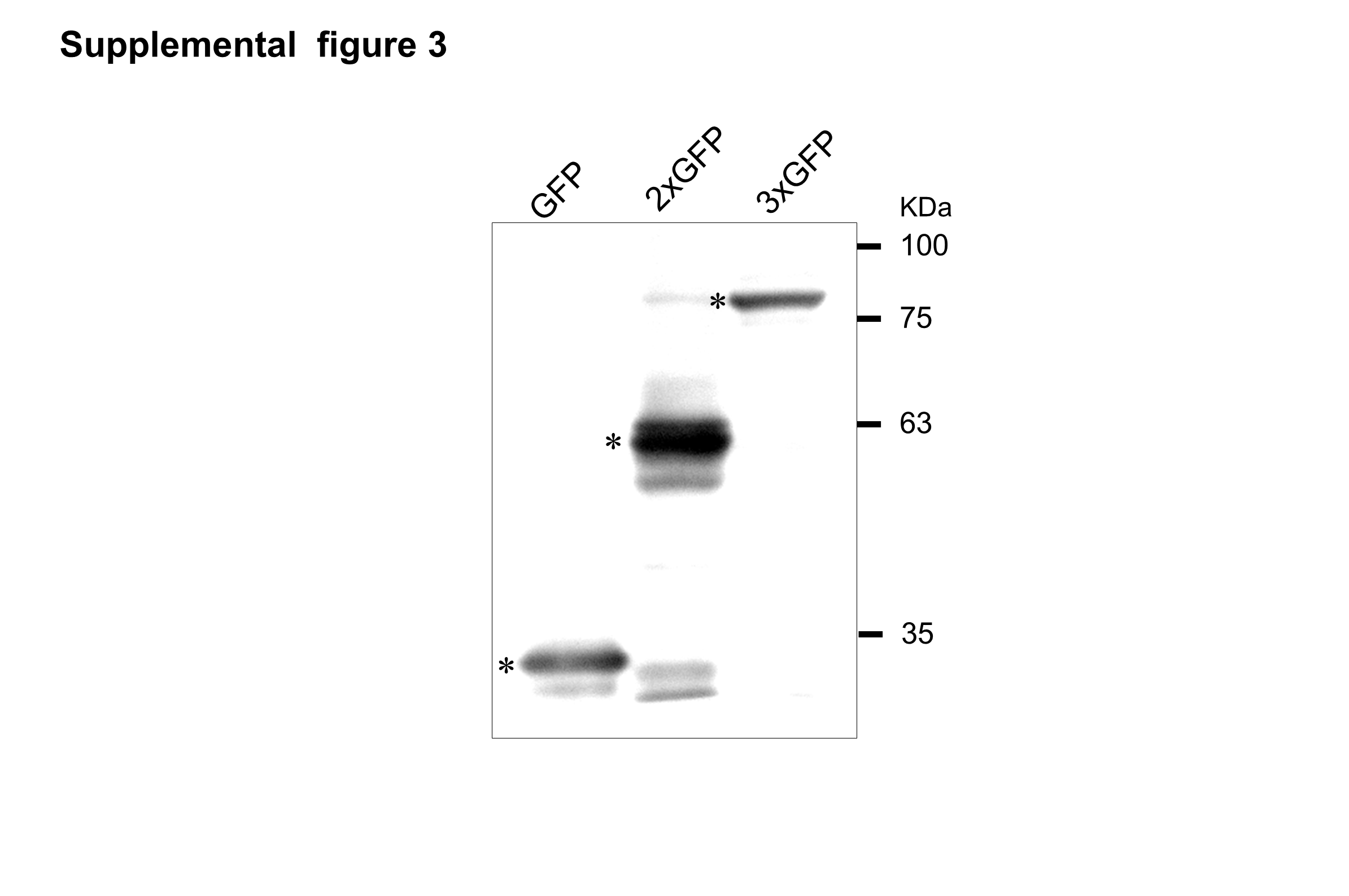

Supplement: Supplementary file 1 [file Image3.TIF]

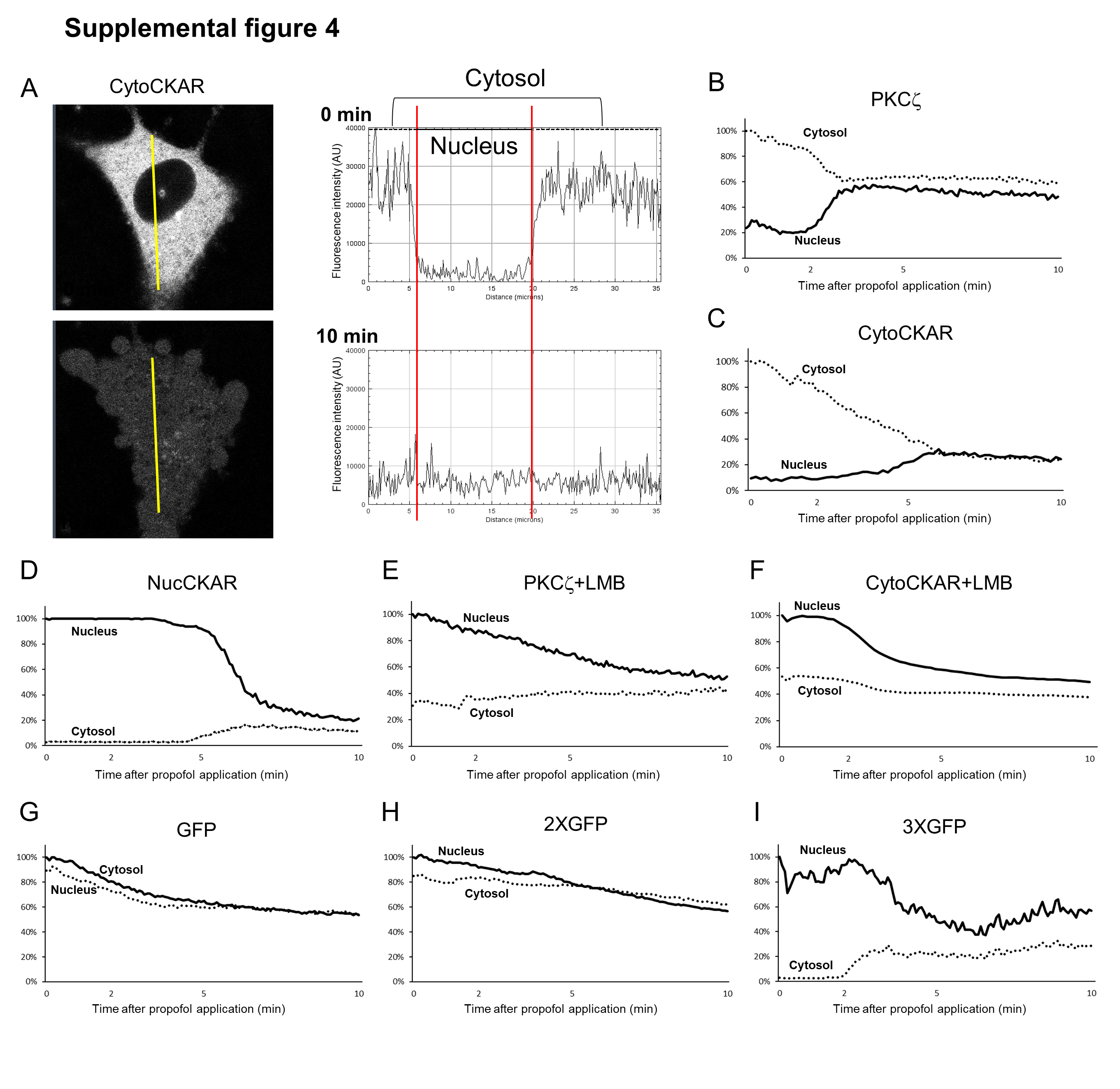

Supplement: Supplementary file 2 [file Image4.TIF]

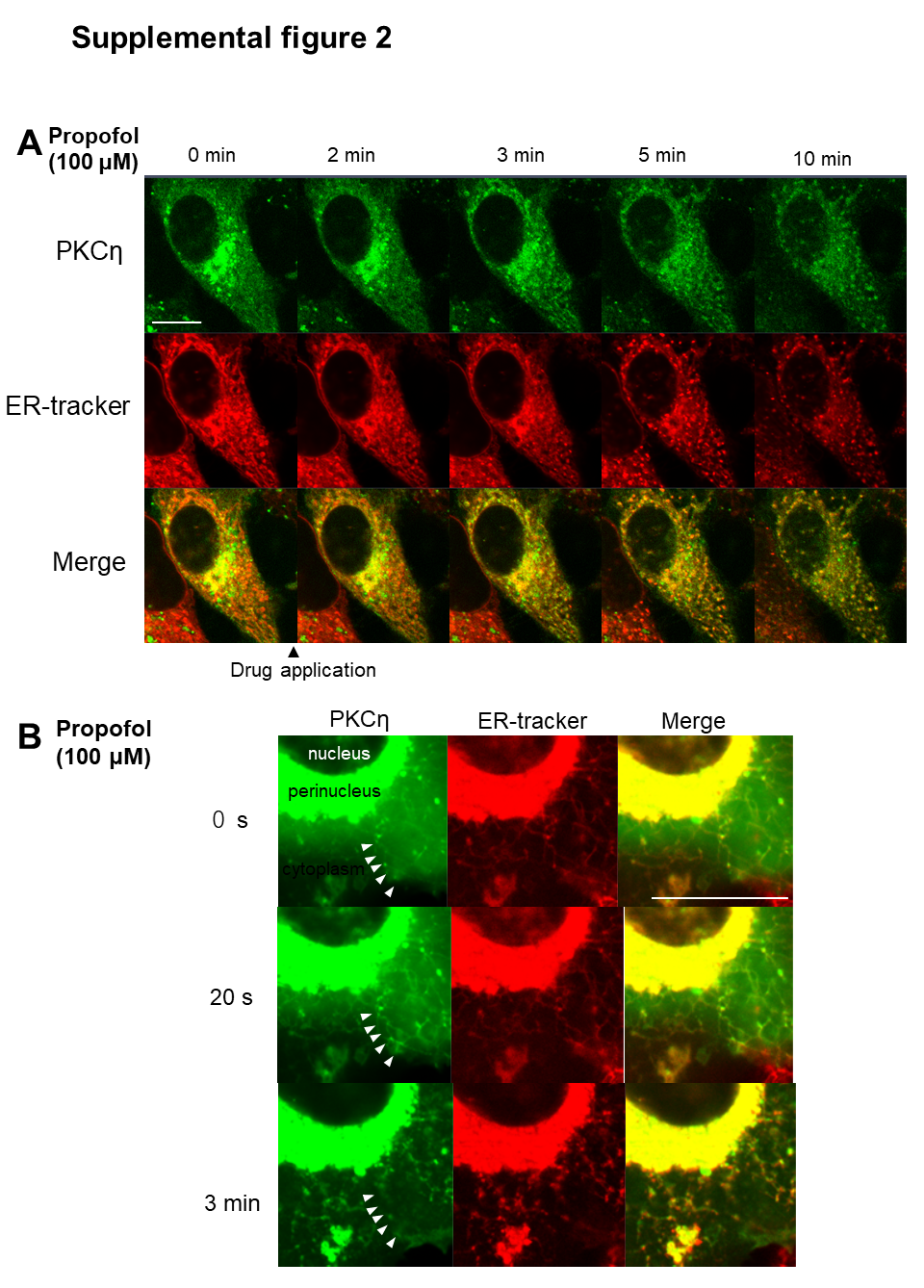

Supplement: Supplementary file 3 [file Image2.TIF]

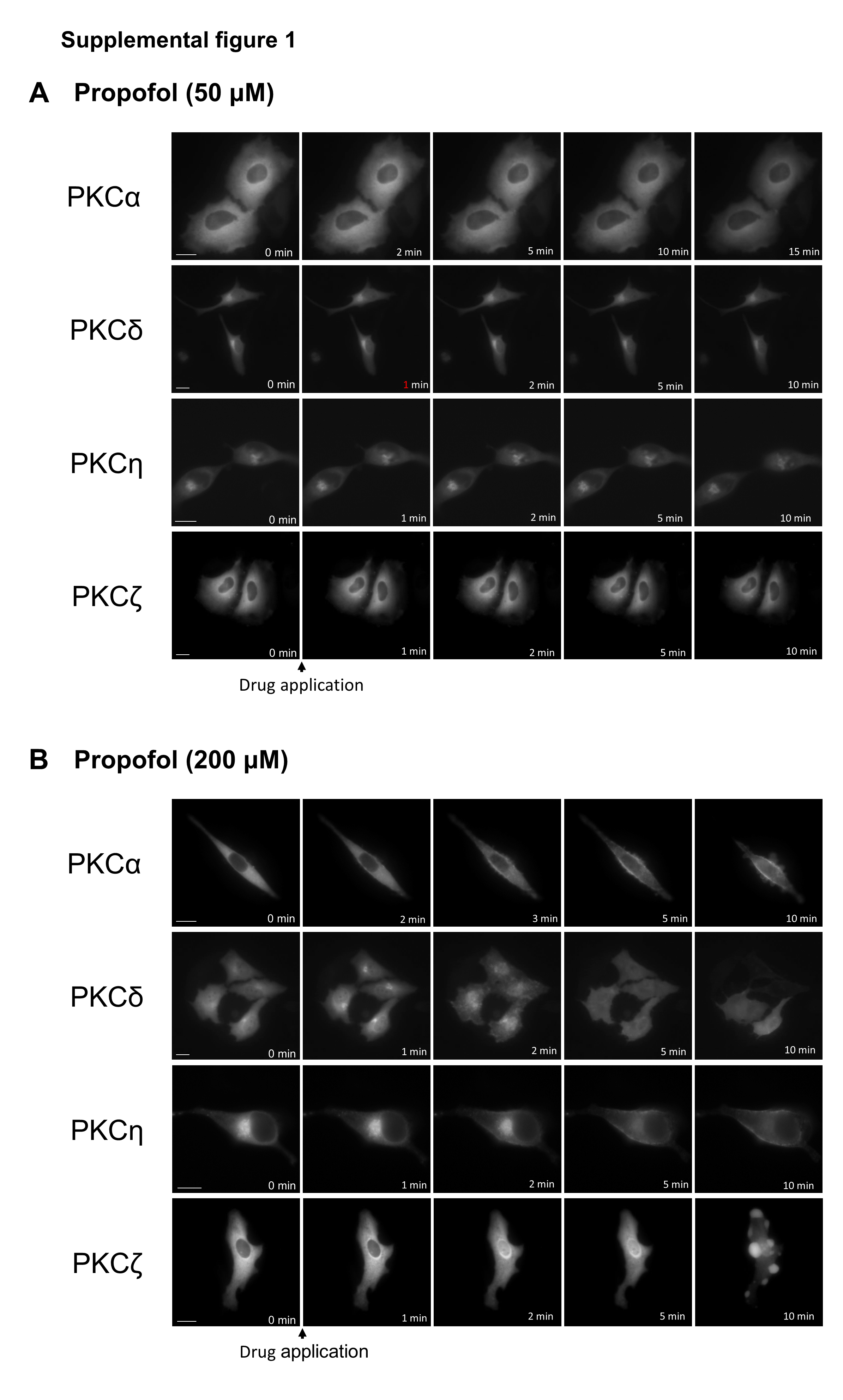

Supplement: Supplementary file 4 [file Image1.TIF]

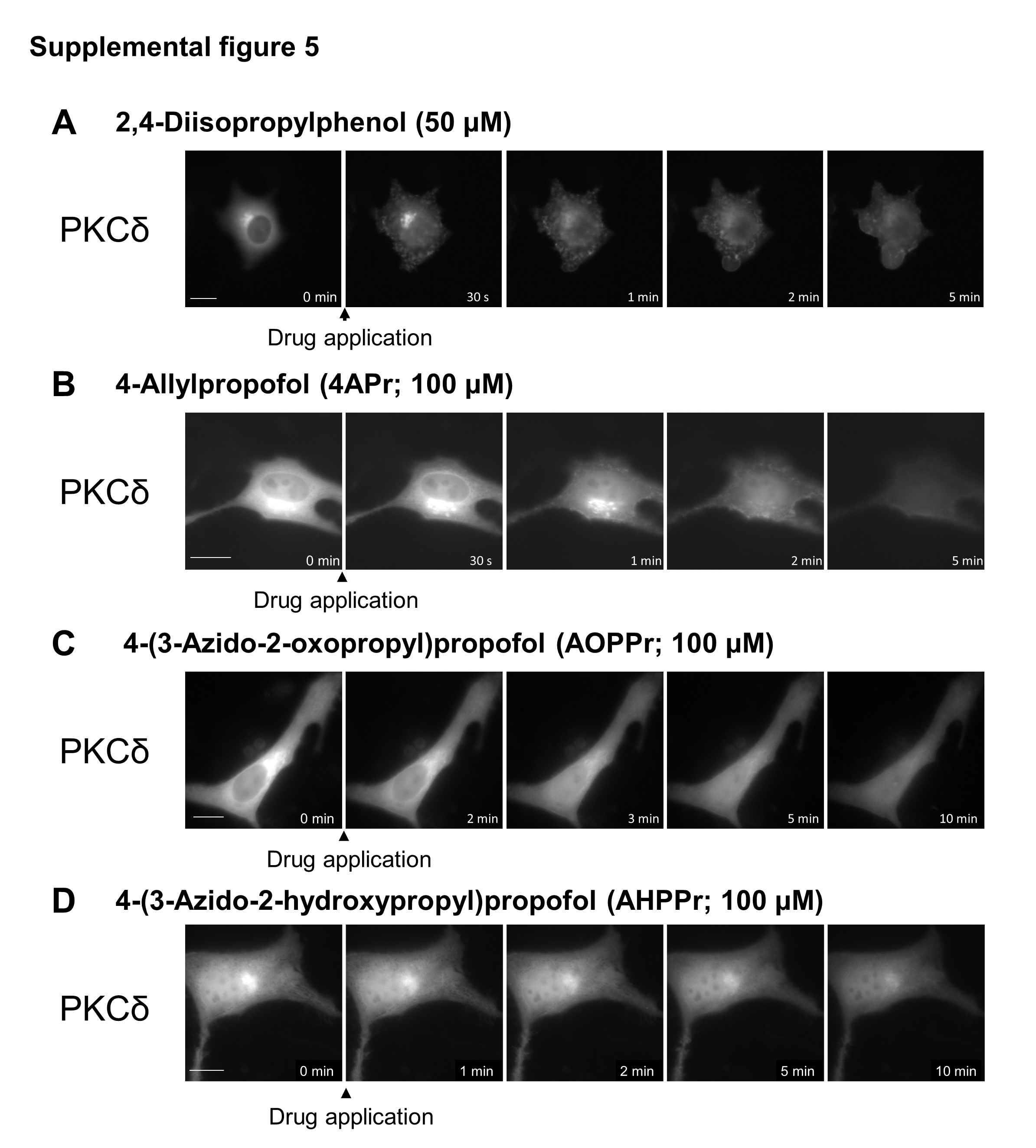

Supplement: Supplementary file 6 [file Image5.TIF]
