# Supplementary material for: Features and mechanisms of propofol-induced protein kinase C (PKC) translocation and activation in living cells
Source: Front Pharmacol. 2023 Nov 7;14:1284586. doi: 10.3389/fphar.2023.1284586 (PMC10662334; doi:10.3389/fphar.2023.1284586)
Supplement: Supplementary file 5 [file DataSheet2.docx]

**Supplementary Figure Legends**

**Supplemental figure 1**

(A) Sequential images of the translocation of green fluorescent protein (GFP)-fused protein kinase C (PKC)-α, PKCδ, PKCη, and PKCζ induced by 50 μΜ propofol. (B) Sequential images of the translocation of　GFP-fused PKCα, PKCδ, PKCη, and PKCζ induced by 200 μM propofol. Images shown in Supplemental Fig. 1 were obtained using a fluorescence microscope. Bars indicate 10 μm.

**Supplemental figure 2**

Simultaneous observation of PKCη-GFP translocation and endoplasmic reticulum (ER) tracker dynamics induced by 100 μM propofol. Images were obtained using a confocal laser-scanning microscope.

(A) Sequential images of propofol (100 μM)-induced PKCη-GFP translocation and ER-tracker dynamics. PKCη-GFP co-localized with the ER tracker before and after propofol application. Propofol altered the localization in ER as dots. PKCη-GFP was also translocated to the accumulated dots in the ER. (B) High-magnification images of PKCη-GFP and ER tracker colocalization. PKCη-GFP is abundantly expressed in the perinuclear ER. PKCη-GFP was also localized in the cytoplasmic reticular ER before the application of propofol (arrowheads, 0 s). Propofol translocated PKCη-GFP to the ER (arrowheads, 20 s). PKCη-GFP was translocated to the ER and accumulated as dots 3 min after application. Bars indicate 10 and 3 μm for A and B, respectively.

**Supplemental figure 3**

Western blotting analysis of singlet GFP and tandem GFPs (2xGFP and 3xGFP). GFP and tandem GFPs were expressed in HeLa cells. Two days after transfection, cell lysates were prepared. Immunoblotting with anti-GFP antibody revealed the molecular sizes of singlet GFP, 2xGFP, and 3xGFP. Asterisks indicate singlet and tandem GFP bands.

**Supplemental figure 4**

Time course of propofol-induced changes in the fluorescence intensity inside and outside the nucleus.

(A) Fluorescence intensity of the line crossing the nucleus was measured using the ImageJ software (Ver.1.46r). Average of the cytoplasmic fluorescence intensity before propofol administration was set to 100%. (B–F) Time course of changes in the average nuclear and cytoplasmic fluorescence intensities of the respective GFP-fused proteins. Abscissa represents the time after propofol administration (min), and the ordinate represents the average fluorescence intensity in the cytoplasm and nucleus (%). Solid lines indicate the changes in fluorescence intensity in the nucleus, and dashed lines indicate the changes in fluorescence intensity in the cytoplasm.

**Supplemental figure 5**

Sequential images of PKCδ-GFP translocation induced by 50 μM 2,4-diisopropylphenol (A), 100 μM 4-allylpropofol (4APr) (B), 100 μM 4-(3-azido-2-oxopropyl)propofol (AOPPr) (C), and 100 μM 4-(3-azido-2-oxopropyl)propofol (AOPPr) (D). Translocation of PKCδ-GFP induced by 2,4-diisopropylphenol and 4APr was similar to that induced by propofol. PKCδ-GFP first accumulated in the Golgi and then translocated to the PM. AOPPr did not translocate PKCδ-GFP to the Golgi and PM but translocated it to the nucleus, which is similar to the propofol-induced translocation of PKCζ-GFP and cyto-CKAR. AOPPr did not induce PKCδ-GFP translocation. Images were captured using a fluorescence microscope. Images in Supplemental figure 5 indicate 2-cells (A), 2-cells (B), 5-cells (C), and 2-cells (D). Bars indicate 10 μm.
