# Supplementary material for: Features and mechanisms of propofol-induced protein kinase C (PKC) translocation and activation in living cells
Source: Front Pharmacol. 2023 Nov 7;14:1284586. doi: 10.3389/fphar.2023.1284586 (PMC10662334; doi:10.3389/fphar.2023.1284586)
Supplement: Supplementary file 7 [file DataSheet1.DOCX]

**Supplemental methodological informations**

**General:**

Commercially available reagents and solvents were used as received, without further purification. Anhydrous solvents (CH_2_Cl_2_, DMF, and THF) were purchased from FUJIFILM Pure Wako Chemicals (Osaka, Japan). Analytical thin-layer chromatography (TLC) was performed on a silica gel 60 F254 plate (Merck) (Darmstadt, Germany). Flash chromatography was carried out using Silica gel 60N (100–210 μm) from Kanto Chemical Co (Tokyo, Japan). Microwave (MW) reaction was carried out using CEM Discover in a sealed tube. IR spectra were recorded on a JASCO FT/IR-4600 spectrophotometer equipped with an NaCl plate. ESI-MS was recorded on a Thermo Fisher Scientific LTQ Orbitrap XL in positive mode. Moreover, ^1^H and ^13^C NMR spectra were recorded on an LA 500 spectrometer (500 MHz for ^1^H and 125 MHz for ^13^C).

1. Experimental details for the synthesis of 4APr (**2**), AHPPr (**6**), and AOPPr (**8**)

2-(Allyloxy)-1,3-diisopropylbenzene (**1**)

To a solution of propofol (2.06 g, 11.6 mmol) in THF (20 mL), NaH (60% in oil, 640 mg, 16.0 mmol) was added portionwise at 0 °C and the mixture was stirred at 0 °C for 30 min. Allyl bromide (1.20 mL, 14.1 mmol) was added dropwise and the mixture was stirred at room temperature (rt) for 5 h. NH_4_Cl (10 mL) was added at 0 °C and the whole was extracted with AcOEt (3 × 10 mL). The organic layers were combined, washed with H_2_O (1 × 10 mL) and brine (1 × 10 mL), and dried over Na_2_SO_4_. The solvent was evaporated *in vacuo* and the residue was purified using CC (hexane only) to give **1** as a colorless oil (1.99 g, 81%). The spectral data are identical to those reported in (1).

(1) El-Qisairi, A. K.; Qaseer, H. A.; Henry, P. M. Oxidation of Olefins by Palladium(II). 18. Effect of Reaction Conditions, Substrate Structure and Chiral Ligand on the Bimetallic Palladium(II) Catalyzed Asymmetric Chlorohydrin Synthesis. *J. Organomet. Chem.* **2002**, *656* (1), 168–176.

4-Allyl-2,6-diisopropylphenol (**2**)

A solution of **1** (300 mg, 1.37 mmol) in Et_2_NPh (2.0 mL) was irradiated under MW at 200 °C for 45 min in a sealed tube. The reaction mixture was then diluted with EtOAc (60 mL). The entire sample was washed with 2 M HCl (1 × 20 mL). The aqueous layer was then extracted using EtOAc (1 × 10 mL). The organic layers were combined, washed with H_2_O (1 × 5 mL) and brine (1 × 5 mL), and dried over Na_2_SO_4_. The solvent was evaporated *in vacuo* and the residue was purified using CC (hexane only to hexane: AcOEt = 6:1) to give **2** as a pale pink oil (212 mg, 71%).

IR (neat) ν_max_ cm^-1^ 3394; ^1^H-NMR (500 MHz, CDCl_3_) δ: 6.87 (2H, s), 5.97 (1H, ddd, *J* = 17.0, 9.9, 6.9 Hz), 5.07 (1H, dd, *J* = 17.0, 1.6 Hz), 5.04 (1H, d, *J* = 9.9 Hz), 4.66 (1H, s), 3.32 (2H, d, *J* = 6.9 Hz), 3.14 (2H, sept, *J* = 6.9 Hz), 1.25 (12H, d, *J* = 6.9 Hz); ^13^C-NMR (125 MHz, CDCl_3_) δ: 148.2, 138.2, 133.6, 131.9, 123.6, 115.2, 40.0, 27.3, 22.8; HRESIMS *m/z* 219.1741 (Calcd for C_15_H_23_O: 219.1749, Δ –3.6 ppm).

5-Allyl-1,3-diisopropyl-2-(methoxymethoxy)benzene (**3**)

To asolution of **3** (1.80 g, 8.25 mmol) in THF (20 mL), NaH (60% in oil, 428 mg, 10.7 mmol) was added portionwise at 0 °C and the whole was stirred at 0 °C for 15 min. MOMCl (0.68 mL, 8.48 mmol) was added and the whole was stirred at rt for 1 h. sat. NH_4_Cl (20 mL) was added at 0 °C and the whole was extracted with AcOEt (3 × 10 mL). The organic layers were combined, washed with H_2_O (1 × 10 mL) and brine (1 × 10 mL), and dried over Na_2_SO_4_. The solvent was evaporated *in vacuo* and the residue was purified using CC (hexane only to hexane: AcOEt = 10:1) to give **3** as a colorless oil (1.94 g, 90%).

IR (neat) no characteristic absorption; ^1^H-NMR (500 MHz, CDCl_3_) δ: 6.91 (2H, s), 5.97 (1H, ddd, *J* = 17.0, 10.0, 6.9 Hz), 5.10 (1H, dd, *J* = 17.0, 1.6 Hz), 5.06 (1H, d, *J* = 10.0 Hz), 4.90 (2H, s), 3.62 (3H, s), 3.34 (2H, d, *J* = 6.9 Hz), 3.22 (1H, sept, *J* = 6.9 Hz), 1.21 (12H, d, *J* = 6.9 Hz); ^13^C-NMR (125 MHz, CDCl_3_) δ: 150.0, 141.6, 137.6, 136.1, 124.0, 115.6, 100.3, 57.3, 40.1, 26.7, 23.9; HRESIMS *m/z* 285.1827 (Calcd for C_17_H_26_NaO_2_: 285.1830, Δ –1.0 ppm).

2-(3,5-Diisopropyl-4-[methoxymethoxy]benzyl)oxirane (**4**)

To a solution of **3** (200 mg, 0.76 mmol) in CH_2_Cl_2_ (1.5 mL), *m*CPBA (65%, 291 mg, 1.10 mmol) and NaHCO_3_ (104 mg, 1.24 mmol) were added at rt and the whole was stirred at rt for 3.5 h. Hexane (10 mL) was added and the precipitates was removed by filtration. The solvent of the filtrate was evaporated *in vacuo* and the residue was purified using CC (hexane: AcOEt = 20:1 to 2:1) to give **4** as a pale yellow oil (173 mg, 82%).

IR (neat) ν_max_ cm^-1^ no characteristic absorption; ^1^H-NMR (500 MHz, CDCl_3_) δ: 6.97 (2H, s), 4.91 (2H, s), 3.62 (3H, s), 3.33 (2H, sept, *J* = 6.9 Hz), 3.13–3.18 (1H, m), 2.75–2.88 (3H, m), 2.58 (1H, dd, *J* = 4.8, 2.5 Hz), 1.22 (12H, d, *J* = 6.9 Hz); ^13^C-NMR (125 MHz, CDCl_3_) δ:150.6, 141.8, 133.5, 124.4, 100.3, 57.3, 52.6, 47.0, 38.7, 26.7, 23.9; HRESIMS *m/z* 301.1774 (Calcd for C_17_H_26_NaO_3_: 301.1780, Δ –1.9 ppm).

1-Azido-3-(3,5-diisopropyl-4-[methoxymethoxy]phenyl)propan-2-ol (**5**)

To a solution of **4** (150 mg, 0.54 mmol) in DMF (1 mL), NaN_3_ (46 mg, 0.71 mmol) was added at rt and the whole was stirred at 90 °C for 1 d. The mixture was partitioned with EtOAc (10 mL) and H_2_O (10 mL), and the aqueous layer was extracted with EtOAc (2 × 10 mL). The organic layers were combined, washed with H_2_O (2 × 1 mL) and brine (1 × 2 mL), and dried over Na_2_SO_4_. The solvent was evaporated *in vacuo* and the residue was purified by CC (hexane: AcOEt = 6:1 to 1:1) to yield **5** as a colorless oil (106 mg, 62%).

IR (neat) ν_max_ cm^-1^ 3450, 2102; ^1^H-NMR (500 MHz, CDCl_3_) δ: 6.93 (2H, s), 4.92 (2H, s), 3.97 (2H, s), 3.69 (2H, s), 3.62 (3H, s), 3.34 (2H, sept, *J* = 6.9 Hz), 2.79 (1H, dd, *J* = 13.7, 5.5 Hz), 2.72 (1H, dd, *J* = 13.7, 8.0 Hz), 1.22 (6H, d, *J* = 6.9 Hz), 1.21 (6H, d, *J* = 6.9 Hz); ^13^C-NMR (125 MHz, CDCl_3_) δ: 150.7, 142.1, 133.2, 124.8, 100.3, 71.8, 57.3, 55.8, 40.7, 26.6, 23.86, 23.85; HRESIMS *m/z* 344.1940 (Calcd for C_17_H_27_N_3_NaO_3_: 344.1950, Δ –2.9 ppm).

4-(3-Azido-2-hydroxypropyl)-2,6-diisopropylphenol (AHPPr, **6**)

To a solution of **5** (11 mg, 0.034 mmol) in CH_2_Cl_2_ (0.5 mL), TFA (0.1 mL, 1.3 mmol) was added at rt and the mixture was stirred at rt for 1 h. The solvent was evaporated *in vacuo* and the residue was purified by CC (hexane only to hexane: AcOEt = 3:1) to give **6** as a colorless oil (5 mg, 53%).

IR (neat) ν_max_ cm^-1^ 3408, 2103; ^1^H-NMR (500 MHz, CDCl_3_) δ: 6.88 (1H, s), 4.75 (1H, s), 3.94-3.99 (1H, m), 3.38 (1H, dd, *J* = 12.6, 3.7 Hz), 3.32 (1H, dd, *J* = 12.6, 6.6 Hz), 3.14 (2H, sept, *J* = 7.1 Hz), 2.76 (1H, dd, *J* = 13.7, 5.3 Hz), 2.70 (1H, dd, *J* = 13.7, 8.0 Hz), 1.26 (12H, d, *J* = 7.1 Hz); ^13^C-NMR (125 MHz, CDCl_3_) δ: 148.9, 134.0, 128.6, 124.3, 71.9, 55.9, 40.6, 27.2, 22.73, 22.72; HRESIMS *m/z* 300.1682 (Calcd for C_15_H_23_N_3_NaO_2_: 300.1688, Δ –2.0 ppm).

1-Azido-3-(3,5-diisopropyl-4-[methoxymethoxy]phenyl)propan-2-one (**7**)

To a solution of **5** (128 mg, 0.40 mmol) in CH_2_Cl_2_ (2.0 mL), Dess-Martin periodinane (DMP) (198 mg, 0.47 mmol) and NaHCO_3_ (166 mg, 1.97 mmol) were added at rt for 1 h. DMP (35 mg, 0.083 mmol) was added at rt the whole was stirred at rt for 1 h. AcOEt (3 mL) was added and the whole was filtered. The solvent of the filtrate was evaporated *in vacuo* and the residue was purified by CC (hexane: AcOEt = 10:1 to 1:1) to give **7** as a colorless oil (95 mg, 75%).

IR (neat) ν_max_ cm^-1^2105, 1728; ^1^H-NMR (500 MHz, CDCl_3_) δ: 6.93 (2H, s), 4.92 (2H, s), 3.97 (2H, s), 3.69 (2H, s), 3.62 (3H, s), 3.34 (2H, sept, *J* = 6.9 Hz), 1.21 (12H, d, *J* = 6.9 Hz); ^13^C-NMR (125 MHz, CDCl_3_) δ: 202.2, 151.4, 142.6, 128.8, 124.9, 100.3, 57.4, 56.6, 47.4, 26.7, 23.8; HRESIMS *m/z* 342.1789 (Calcd for C_17_H_25_N_3_NaO_3_: 342.1794, Δ –1.5 ppm).

1-Azido-3-(4-hydroxy-3,5-diisopropylphenyl)propan-2-one (AOPPr, **8**)

To a solution of **7** (9 mg, 28 μmol) in CH_2_Cl_2_ (0.5 mL) TFA (0.1 mL, 1.3 mmol) was added at rt and the mixture was stirred at rt for 1 h. The solvent was evaporated *in vacuo* and the residue was purified by CC (hexane: AcOEt = 10:1 to 3:1) to give **8** as a colorless oil (7 mg, 90%).

IR (neat) ν_max_ cm^-1^ 3512, 2105, 1718; ^1^H-NMR (500 MHz, CDCl_3_) δ: 6.87 (2H, s), 3.98 (2H, s), 3.67 (2H, s), 3.14 (2H, sept, *J* = 6.9 Hz), 1.25 (12H, d, *J* = 6.9 Hz); ^13^C-NMR (125 MHz, CDCl_3_) δ: 202.8, 149.4, 134.4, 124.42, 124.37, 56.6, 47.4, 27.2, 22.7; HRESIMS *m/z* 298.1526 (Calcd for C_15_H_21_N_3_NaO_2_: 298.1531, Δ –1.7 ppm).
